# Optimal *H*_∞_ Control for Lateral Dynamics of Autonomous Vehicles

**DOI:** 10.3390/s21124072

**Published:** 2021-06-13

**Authors:** Gianfranco Gagliardi, Marco Lupia, Gianni Cario, Alessandro Casavola

**Affiliations:** Dipartimento di Ingegneria Elettronica, Informatica e Sistemistica (DIMES), Universitá della Calabria, 87036 Rende, CS, Italy; mlupia@dimes.unical.it (M.L.); gcario@dimes.unical.it (G.C.); a.casavola@dimes.unical.it (A.C.)

**Keywords:** autonomous vehicles, automotive control, *H*_∞_ control, lateral control, linear matrix inequalities, path tracking, steering angle control

## Abstract

This paper presents the design and validation of a model-based $H_{\infty }$ vehicle lateral controller for autonomous vehicles in a simulation environment. The controller was designed so that the position and orientation tracking errors are minimized and so that the vehicle is able to follow a trajectory computed in real-time by exploiting proper video-processing and lane-detection algorithms. From a computational point of view, the controller is obtained by solving a suitable LMI optimization problem and ensures that the closed-loop system is robust with respect to variations in the vehicle’s longitudinal speed. In order to show the effectiveness of the proposed control strategy, simulations have been undertaken by taking advantage of a co-simulation environment jointly developed in Matlab/Simulink *©* and Carsim 8 *©*. The simulation activity shows that the proposed control approach allows for good control performance to be achieved.

## 1. Introduction

Vehicle safety is a major human challenge. The World Health Organization (WHO) reports more than one million fatalities in traffic accidents and around 20–50 million injuries each year worldwide. The WHO estimates that approximately one-half of the fatalities involve what are referred to as “vulnerable road users” i.e., pedestrians, cyclists, and motorbikes [1]. A general system approach that accounts for the interactions between humans, vehicles, and the environment and provides the necessary countermeasures in preventing crashes is required to tackle this problem.

With respect to this problem, the automotive industry has made relevant strides, and at the same time, governments around the world have increased safety regulations. Passive and active systems have been developed throughout the years to enhance vehicle safety [2]. Moreover, further research efforts have been devoted to investigating the main causes of road accidents and to designing mathematical models in charge of predicting and reducing the damage caused by road accidents on the basis of statistical analysis of “correlate factors” such as driver gender and age, alcohol use, vehicle type, etc. [3,4]. In recent years, research on Advanced Driving Assistant System (ADAS) and intelligent vehicles have attracted a lot of attention due to technological progress in the fields of sensing, communication, and information processing. This interest pertains not only to the intelligent functions that support a driver during driving but also to automated driving. As road accidents occur frequently and result in property damages, injury, and even death, all of which impose a high cost to societies, the main aim in developing new and more sophisticated functions for ADAS systems is to improve road safety, to mitigate traffic issues, and to improve driving comfort [5,6].

ADAS can be viewed as real-time systems able to react quickly to multiple inputs and to prioritize incoming information to prevent accidents. ADAS can be categorized and divided into six levels based on the amount of automation. This scale is provided by the Society of Automotive Engineers (SAE) and has been designed to clarify and simplify the SAE standard J3016 of *Levels of Driving Automation*: the standard defines six levels of driving automation, from SAE Level 0 to SAE Level 5 [7,8]. In Level 0 (*No Driving Automation*), ADAS cannot control the car and can only provide information for the drivers to interpret the current situation on their own: parking sensors, traffic signs recognition, lane departure warning systems, etc. belong to this level. Levels 1 and 2 are very similar in that they both let the driver make most of the decisions. The difference is that, at Level 1 (*Driver Assistance*), the ADAS can take over the control of one functionality (e.g., adaptive cruise control, emergency brake assist, etc.). On the other hand, at Level 2 (*Partial Driving Automation*), the ADAS can take over the control of multiple functionalities to aid the driver: autonomous obstacle avoidance, autonomous parking, etc. At Level 3 (*Conditional Driving Automation*), vehicles have “environmental detection” capabilities and can make informed decisions for themselves, such as accelerating past a slow-moving vehicle. However, they still require human override. The driver must remain alert and ready to take control if the system is unable to execute the task. The key difference between Level 3 and Level 4 is that, at Level 4 (*High Driving Automation*), drivers can intervene if things go wrong or there is a system failure. In this sense, these cars do not require human interaction in most circumstances. However, a human still has the option to manually override the system. At level 5 (*Full Driving Automation*), vehicles do not require human attention: the “dynamic driving task” is eliminated.

In this respect, lane-detection (LD) and lane-keeping (LK) systems are important challenges. An autonomous driving car can be viewed as a vehicle that is able to drive in different driving scenarios by replacing human actions at different levels. Then, autonomous cars must be equipped with all of the necessary functionalities to solve three main tasks [9]:

- **Environmental perception**. Due to the fact that the environment in which cars are used must be considered partially unknown, a vision system (cameras and sensors such as radar, lidar, etc.) must be used to detect road boundaries, various objects (e.g., obstacles and pedestrians), and other vehicles. In this way, it is possible to provide a dynamic map of the environment around the autonomous vehicle.
- **Trajectory generation**. This task concerns the generation of a reference trajectory (or reference path) in the navigable environment.
- **Vehicle control**. This task consists of designing control algorithms for longitudinal and lateral control, which use available actuators (accelerator pedal, brakes, steering wheel, etc.) to track the reference trajectory.

In order to achieve these goals, the autonomous vehicle has to use a number of well-placed sensors that detect and continuously observe the location and movement of other vehicles, people, traffic lights, etc.

This paper focuses on the second and third items of the aforementioned steps and relies on the implementation of a lateral controller that automatically acts on the steering wheel to track a reference trajectory. It is important to note that the design of the vehicle’s longitudinal controller is beyond the scope of this work. Observe in fact that the later and longitudinal dynamics are practically decoupled so that the longitudinal controller can be designed separately.

Due to the high non-linearity of the system, the uncertainty in the model parameters, and the presence of disturbances, robustness of the controller is an important goal. There is rich literature that reports relevant research efforts to provide suitable lateral guidance in autonomous vehicles. Beside standard (PI-PID) control approaches [10,11,12,13,14], approaches based on Model Predictive Control (MPC) have attracted considerable attention in trajectory following applications. In [15], an MPC algorithm that is in charge of generating smooth and collision-free trajectories for a given predicted velocity profile was presented. In [16], an MPC controller was designed so that the front steering angle is computed in order to follow the trajectory on slippery roads at the highest possible entry speed. The MPC controller was designed by assuming that the trajectory is known over a finite horizon. A model predictive controller that uses a speed-dependent adaptation of the prediction model and cost function weights to ensure a stable and precise path tracking performance was presented in [17]. An important issue was related to the fact that the performance of a path tracking controller can be affected by system malfunctions due to internal factors (e.g., sensors and electronic device faults) or to the road (e.g., marking quality, etc.), light, and weather conditions. In this respect, in [18,19], the LD system performance and probability of fault were investigated with special focus on the effects of the physical infrastructure related to road characteristics and conditions. In [20], a robust fault-tolerant path tracking control algorithm based on adaptive MPC was proposed. The main drawback in the use of MPC control strategies was in the computation time that, for high-speed driving, becomes too large for real-time operations. Other control strategies have also been used for lateral control purposes: a $H_{\infty }$ robust lateral controller for differential GPS-based autonomous vehicle was adopted in [21] whilst a $H_{\infty }$ path-tracking control problem of network-based autonomous vehicles was presented in [22]. Specifically, the controller has been shown to be robust with respect to parameter uncertainties and external disturbances and to allow for good performance in the presence of delays and packet dropout. In [23] and in [9], Linear Quadratic Regulator (LQR) and sliding mode control approaches were presented, respectively.

In this paper, the main goal is to design a control system able to perform the trajectory following task for an autonomous vehicle. The control architecture accounted for is modular and exploits information from onboard sensors and vision systems. The designed control architecture is in charge of the following:

- Determining a reference trajectory in real-time on the basis of the output of a lane-detection procedure that elaborates the environment information acquired by a camera and
- Allowing for path following by controlling the steering angle.

A particularly complex problem arises when the road strips are incomplete or totally missing and, hence, the camera is unable to suitably define the boundaries of the driving lane (e.g., country roads lacking suitable road signage, vehicle/queue management when approaching toll gates or motorway connections, etc.). Furthermore, another important issue is guaranteeing the performance of the overall control system at various vehicle speeds.

In this respect, this paper proposes a control architecture that is capable of verifying if the lane-detection procedure is able to provide a lane estimation and, at the same time, to provide a control action robust with respect to disturbances and variation in the vehicle speed. The lane-detection procedure is designed according to [24], and the control action exploits an optimal $H_{\infty }$ tracking controller that is computed by solving a convex LMI optimization problem [25].

The paper is organized as follows. Section 2 describes the steering control architecture and all of its subsystems: the lane-detection and the trajectory-generation modules and the model of the vehicle lateral dynamics. In Section 3, the robust $H_{\infty }$ lateral controller is presented, whilst in Section 4, the simulation results regarding two driving scenarios are reported. Some conclusions end the paper.

## 2. Steering Control for Autonomous Vehicles

**Figure 1:**
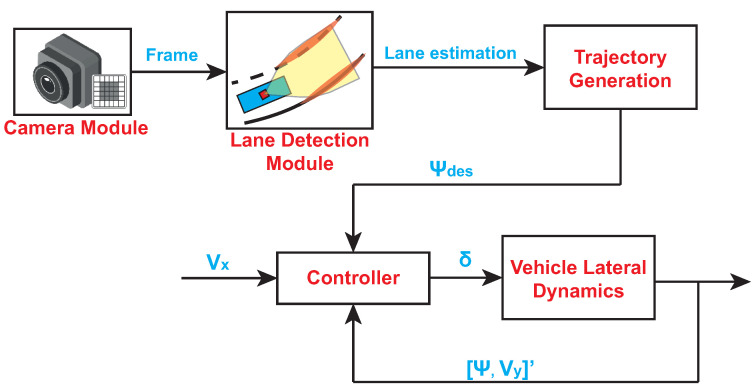
Steering control schematic.

Steering control for autonomous vehicles is a control architecture that combines various active safety systems and allows for a vehicle to follow a path computed in real-time on the basis of the road conditions (Figure 1):

- **Camera module**, which records the current view of the road. The camera is directed towards the front of the vehicle;
- **Lane-detection module**, which is in charge of detecting lane strips in an image. It makes use of the detected strips in order to compute an estimation of the car position within the lane;
- **Trajectory-generation module**, which is in charge of computing the reference trajectory ($\psi_{des}$) on the basis of the lane estimation provided by the lane-detection module;
- **Controller**, which provides the necessary control actions to guarantee that the vehicle follows the reference trajectory; and
- **Vehicle lateral dynamics**, which is the module that implements the mathematical model of lateral vehicle motion.

By taking into account the steering control schematic reported in Figure 1, the design procedure was accomplished by considering two main tasks:

1. Designing and testing a video-processing algorithm in charge of providing an estimation of the car position within the lane in a real application context and
2. Designing and validating a steering controller that allows the car to follow a reference trajectory in a co-simulation environment.

In what follows, all of the devices and modules that pertain to the steering control architecture are briefly described.

### 2.1. Camera Module

An autonomous vehicle can be equipped with various types of sensors (e.g., radar, lidar, camera, etc.) located anywhere outside or inside the vehicle [1].

**Figure 2:**
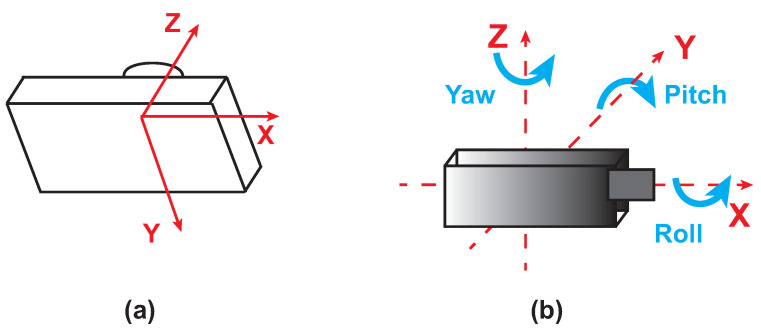
Camera module: reference coordinates (**a**) and ISO convention (**b**).

**Figure 3:**
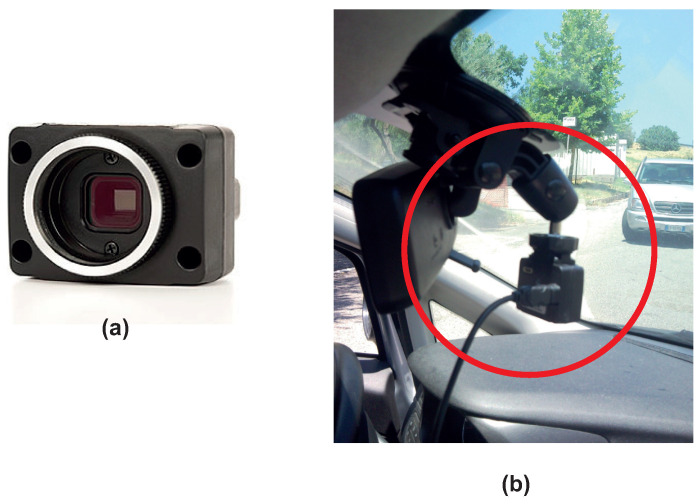
CMOS FireFly MV camera (**a**) and camera vehicle mounting (**b**).

The monocular camera is a standard vision sensor used in automated driving applications. This type of sensor can be useful in object- and lane-boundary detection and in object-tracking applications. As highlighted in Figure 2a, the camera coordinate system is described by a standard Cartesian representation with the origin located at the cameras’ optical center. On the other side, the yaw, pitch, and roll angles are referenced according to the ISO convention (refers to Figure 2b). The camera used in our application is a CMOS FireFly MV camera (Figure 3a) [26,27]. Figure 3b shows the installation of the camera on the car windshield. Note that, in the setup of the camera system, the parameters reported in Table 1 were accounted for. The camera, with a specified rate, acquired the frames that were outputted to the lane-detection module.

### 2.2. Lane-Detection and Trajectory-Generation Modules

**Figure 4:**
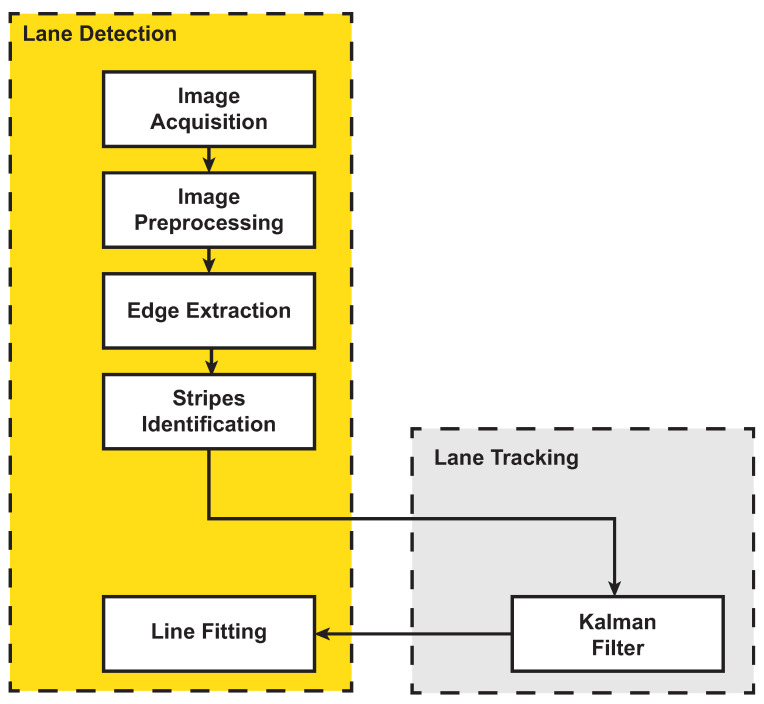
Lane-detection and tracking algorithm.

The lane-detection module is in charge of estimating the strips and road lanes. The typical steps for lane strip identification are reported in Figure 4: the camera acquires an image frame, and a preprocessing module in charge of improving the quality of the frame starts (e.g., improves the image contrast, reduces the image noise, etc.). Then, the processed image is further elaborated to extract the contours and to identify the pixels belonging to the lane strips. A Kalman filter is used to predict and track the lane strips over consecutive frames.

Finally, a fitting process procedure is accomplished to aggregate different groups of pixels potentially belonging to the same strip and to provide the strip coordinates and an estimate of the curvature. The strip-fitting problem could be made more efficient if one knows that certain forms are present in the image and a good mathematical model (e.g., linear, linear parabolic, polynomial, clothoid, and spline models) is available to describe them. Here, lane-strip determination is based on a linear–parabolic (LP) fitting [28].

**Figure 5:**
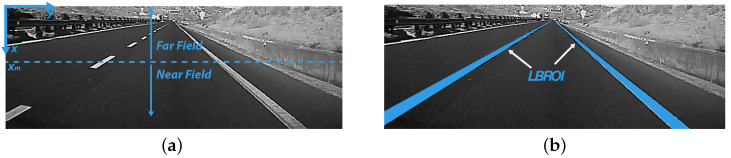
Linear–parabolic fitting: near and far field image separation (**a**) and LBROI (**b**).

The basic idea is to separate the frame into two fields, near field and far field (Figure 5a), using a fixed threshold $x_{m}$ and to perform a linear fitting in the first one and a quadratic fitting in the second one. The curve that represents the strips is given by the following:

(1) $$f\left(x\right)=\left\{\begin{array}{cc} a+b(x-x_{m}) & x>x_{m}\\ a+b\left(x-x_{m}\right)x+c{\left(x-x_{m}\right)}^{2} & x\leq x_{m} \end{array}\right.$$

In order to accomplish such an LP fitting, the first step is to identify the two areas of interest (Lane Boundary Region of Interest (LBROI)) within the current frame (Figure 5b).

The parameters *a*, *b*, and *c* of Equation (1) are determined by a linear fitting minimizing the weighted quadratic error *E*:

(2) $$E=\sum_{i=1}^{m}M_{n_{i}}{\left[y_{n_{i}}-f\left(x_{n_{i}}\right)\right]}^{2}+\sum_{j=1}^{n}M_{f_{j}}{\left[y_{f_{j}}-f\left(x_{f_{j}}\right)\right]}^{2}$$

where $\left(x_{n_{i}},y_{n_{i}}\right)$, with i=1,…,m, represents the *m*th pixel coordinates and where $M_{n_{i}}$ represents the corresponding magnitudes (only for the pixels ≠0). Similarly, $\left(x_{f_{j}},y_{f_{j}}\right)$ and $M_{f_{j}}$, with j=1,…,n, represent the same quantities for the *n* pixels in the far field with magnitudes ≠0.

The *E* error is minimized analytically by finding a solution to the following linear system of equations:

(3) $$A^{T}WAC=A^{T}WB$$

where

(4) $$A=\left[\begin{array}{ccc} 1 & x_{n_{1}}-x_{m} & 0\\ \vdots & \vdots & \vdots \\ 1 & x_{n_{m}}-x_{m} & 0\\ 1 & x_{f_{1}}-x_{m} & {\left(x_{f_{1}}-x_{m}\right)}^{2}\\ \vdots & \vdots & \vdots \\ 1 & x_{f_{n}}-x_{m} & {\left(x_{f_{n}}-x_{m}\right)}^{2} \end{array}\right]\;,\;W=\left[\begin{array}{cccccc} M_{n_{1}} & \; & & \; & & \\ \; & \ddots & \; & & \; & \\ \; & \; & M_{n_{m}} & \; & & \\ \; & \; & & M_{f_{1}} & \; & \\ \; & \; & & \; & \ddots & \\ \; & \; & & \; & & M_{f_{n}} \end{array}\right]$$

(5) $$C=\left[a,b,c^{T}\right]\;,\;B=\left[y_{n_{1}},\ldots ,y_{n_{m}},y_{f_{1}},\ldots ,y_{f_{n}}\right]$$

The procedure is applied to both strips of the lane.

**Figure 6:**
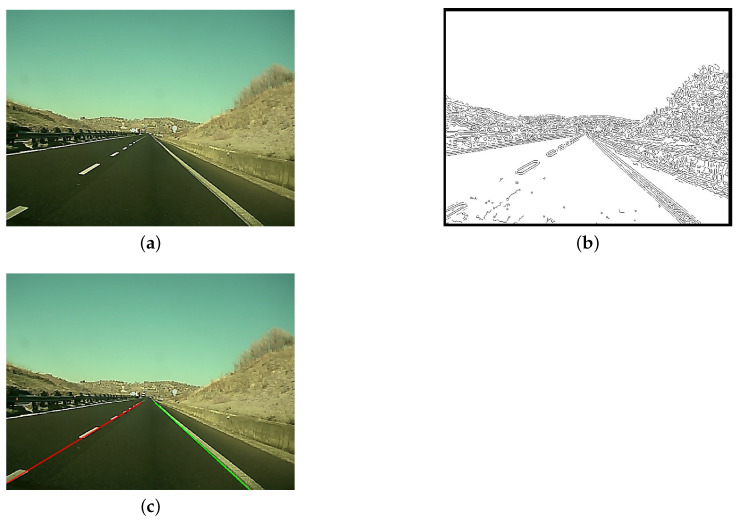
Lane detection: image acquisition and preprocessing (**a**), edge extraction (**b**), and line identification (**c**).

In Figure 6, the various phases of the lane-detection process are shown on a real road frame.

Details about the implementation of the lane-detection algorithm can be found in [24].

**Figure 7:**
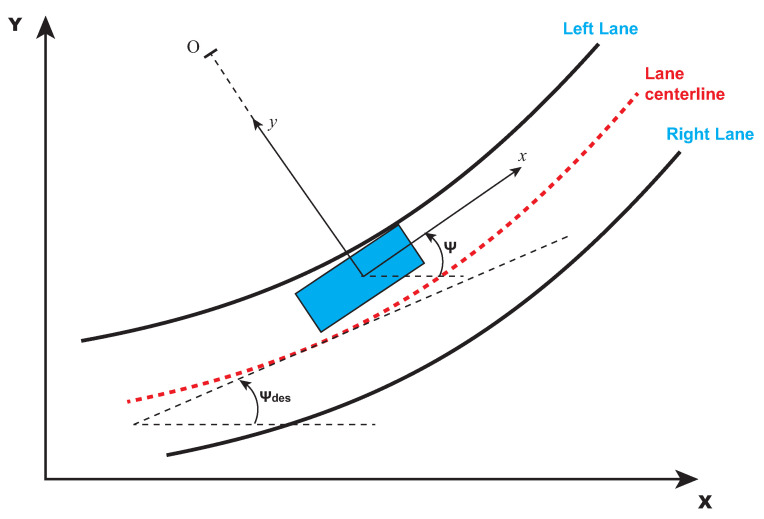
Road representation used in lateral control, highlighting the lane centerline, and the left and right lanes.

At the end of the lane-detection procedure, the trajectory-generation computation task starts. The trajectory-generation phase consists of finding the trajectory and of computing its curvature on the basis of the information on the lane strips coming from the previous step. In general, the reference trajectory consists of the curvature of the lane centerline (refers to Figure 7) and can be computed as the average value between the left strip of the lane and the right one. Furthermore, since the controller needs to receive the necessary information to generate the control action, the computed curvature needs to be recast in terms of the desired yaw angle ($\psi_{des}$). In this respect, the following are highlighted [29]:

- The reference angular velocity of the vehicle can be defined as ${\dot{\psi }}_{des}=V/R$, where *V* is the vehicle speed at the center of gravity and *R* is the radius of curvature.
- The radius of curvature *R* can be computed as the inverse of the absolute value of the curvature *k* at a point: $R=1/\left|k\right|$.

**Figure 8:**
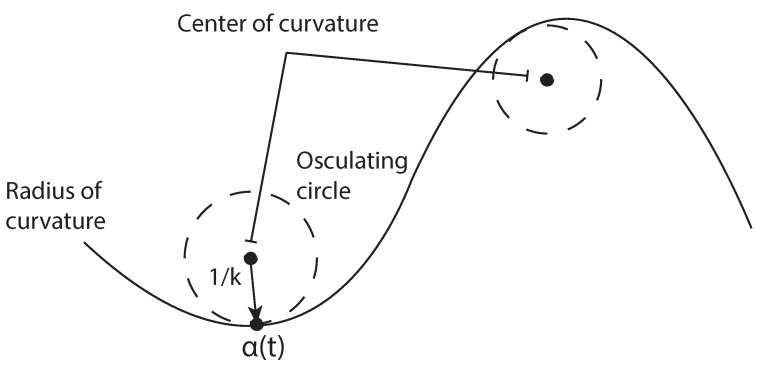
Osculating circle and radius of curvature.

With reference to Equation (1) and Figure 8, in order to compute the curvature *k*, it is necessary to consider the *osculating circle*, that is, the circle with radius *R* centered at the curvature center. This circle allows one to locally approximate the curve up to the second order. Then, the curvature *k* can be expressed in terms of the first- and second-order derivatives of the curve *f* as [30]:

(6) $$k=\frac{\left|\ddot{f}\right|}{{\left[1+{\dot{f}}^{2}\right]}^{3/2}}$$

Then, the desired yaw angle can be computed as follows:

(7) $$\psi_{des}=\int_{0}^{t}V\left|k\right|dt$$

It is important to note that, due to the fact that it is preferable that the vehicle always stays at the lane centerline, this task plays a key role in the overall steering control process. Since the lane detection algorithm (Algorithm 1) can fail and the detection algorithm cannot perform correct lane-strip identification, four cases must be accounted for in the reference trajectory computation:

1. Both the left and right strips are detected.
2. Only the left strip is detected.
3. Only the right strip is detected.
4. The left and right strips are not found: in this case, a safe driving condition (*limp home* driving mode) must be activated in order to avoid dangerous events from taking place.

Then, the following logic is included in the computation of the curvature *k* for the trajectory-generation procedure where *r*, $k_{l}$, and $k_{r}$ are a constant indicating the half-lane dimension (*r* = 1.8 [m]) and the curvature of the left and right strips, respectively.
**Algorithm 1** Curvature computation
1: **procedure** CURVATURE(*k_l_*, *k_r_*)
2:  **if**
*k_l_* & *k_r_*
**then**         ▹ Both the left and right strips are detected.
3:   $k=(k_{l}+k_{r})/2$
4:  **else if**
$k_{l}\;\;\&\;\;!k_{r}$
**then**           ▹ Only the left strip is detected.
5:   $k=k_{l}/\left(1-k_{l}*r\right)$
6:  **else if**
$k_{r}\;\;\&\;\;!k_{l}$
**then**          ▹ Only the right strip is detected.
7:   $k=k_{r}/\left(1+k_{r}*r\right)$
8:  **else**               ▹ The left and right strips are not found.
9:    *disable autonomous guidance and activate “limp home” strategy*
10: **end if**
11: **end procedure**


**Figure 9:**
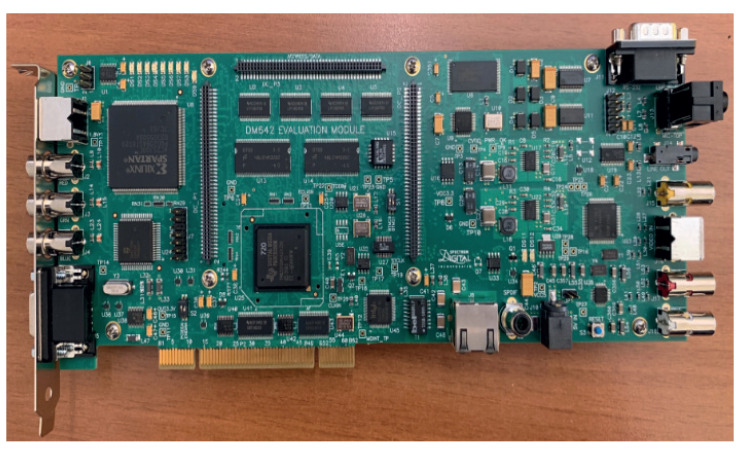
Board EVM TMS320DM642.

The lane-detection and trajectory-generation algorithms were implemented via the Image Processing and Computer Vision Toolbox available in Matlab/Simulink. In order to build up a prototypal setup, the Matlab/Simulink code of the video-processing algorithm was coded in the *C language*, suitable for the Board EVM TMS320DM642. This evaluation module (Figure 9) is a low-cost standalone development platform that enables users to evaluate and develop applications for the TI C64xx Digital Signal Processor (DSP) family [31].

### 2.3. Vehicle Lateral Dynamics

In the design of a model-based lateral controller for autonomous cars, knowledge of the car model plays a key role in the design of the path planning and path-tracking modules. Under certain assumptions (e.g., for low longitudinal speed of the vehicle), a kinematic model can be considered for vehicle lateral motion. When this type of modeling approach is adopted, it is possible to provide a mathematical description of the vehicle motion without considering the forces that influence the motion. The motion equations are simply based on geometric relationships.

**Figure 10:**
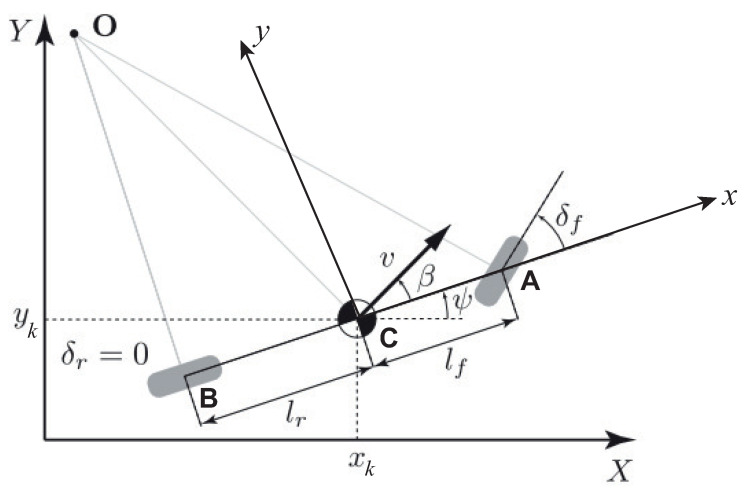
Vehicle bicycle model.

In order to compute the motion equations for the kinematic model, the bicycle model reported in Figure 10 is usually used [32]. In this figure, two coordinate systems are highlighted: the *world coordinate* system (X,Y) and the body-fixed coordinate system (x,y). The major assumption in the correct use of the kinematic model is that the velocities at the points **A** and **B** are always oriented in the direction of the wheels’ orientation, i.e., this corresponds to assuming that the wheel slip angles are zero, a condition that almost holds true at low speeds. The total lateral force needed to ride a circular road of radius **R** is given by the following:

(8) $$F_{y}=\frac{mv^{2}}{R}$$

where *m* and *v* are the vehicle mass and the vehicle speed, respectively. The motion equations for the kinematic model are given by the following [29]:

(9) $$\left\{\begin{array}{ccc} {\dot{x}}_{k} & = & vcos(\psi +\beta )\\ {\dot{y}}_{k} & = & vsin(\psi +\beta )\\ {\dot{\phi }}_{z} & = & \frac{vcos(\beta )}{l_{f}+l_{r}}\left(tan\left(\delta_{f}\right)-tan\left(\delta_{r}\right)\right) \end{array}\right.$$

where

- $x_{k}$ and $y_{k}$ are the trajectories;
- $\phi_{z}$ is the yaw angle;
- ψ is the vehicle orientation;
- β is the slip angle;
- $l_{f}$ and $l_{r}$ are the distances of points **A** and **B** from the center of gravity (**C**), respectively; and
- $\delta_{f}$ and $\delta_{r}$ are the front and rear wheels steering angles, respectively. Note that the steering angle of rear wheel is assumed to be $\delta_{r}=0$.

It is important to note that the kinematic model (9) is not accurate at higher longitudinal vehicle speeds due to the fact that the previous assumption is no longer true. In this case, a dynamic model of the lateral vehicle model should be accounted for.

The dynamic model can be derived by taking into account the two-degrees-of-freedom bicycle model depicted in Figure 10, where the degrees of freedom are represented by the vehicle lateral position (*y*) and the vehicle yaw angle (ψ). Then, starting from Newton’s second law for motion along the *y*-axis and by computing the moment balance around the vertical *z*-axis, the following model can be derived for the vehicle lateral dynamics:

(10) $$\left\{\begin{array}{ccc} ma_{y} & = & F_{yf}+F_{yr}\\ I_{z}\ddot{\psi } & = & l_{f}F_{yf}-l_{r}F_{yr} \end{array}\right.$$

in which

- $a_{y}$ is the inertial acceleration of the vehicle at the center of gravity in the *y*-axis direction;
- $F_{yf}$ and $F_{yr}$ are, respectively, the lateral tire forces of the front and rear wheels; and
- $I_{z}$ is the inertia of the vehicle around the *z*-axis.

**Figure 11:**
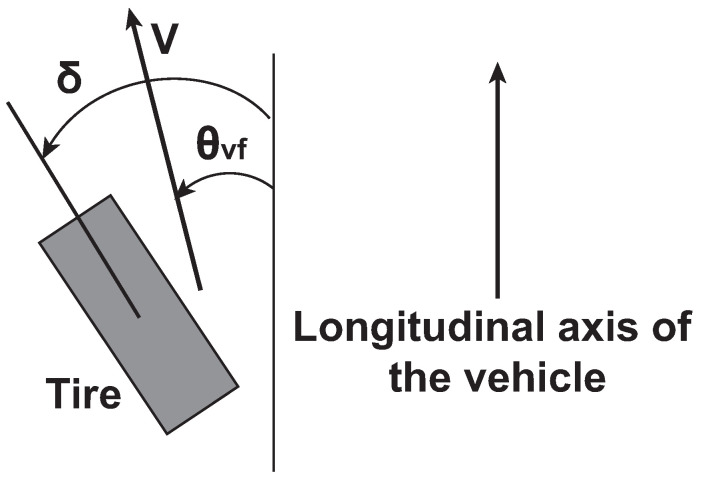
Slip angle.

In Equation (10), two terms contribute to the lateral acceleration ($a_{y}$): the acceleration due to the motion along the *y*-axis ($\ddot{y}$) and the centripetal acceleration ($v_{x}\dot{\psi }$). Furthermore, the lateral tire forces are proportional to the slip angle that is defined as the angle between the orientation of the tire and the orientation of the velocity vector of the wheel (see Figure 11).

Then, Equation (10) can be written as follows:

(11) $$\left\{\begin{array}{ccc} m(\ddot{y}+\dot{\psi }v_{x}) & = & 2C_{\alpha f}\left(\delta -\theta_{vf}\right)+2C_{\alpha f}\left(-\theta_{vr}\right)\\ I_{z}\ddot{\psi } & = & l_{f}2C_{\alpha f}\left(\delta -\theta_{vf}\right)-l_{r}2C_{\alpha f}\left(-\theta_{vr}\right) \end{array}\right.$$

Furthermore, by assuming small-angle approximation [29],

$$\theta_{vf}\approx \frac{\dot{y}-l_{f}\dot{\psi }}{v_{x}},\;\;\;\;\;\;\theta_{vr}\approx \frac{\dot{y}-l_{r}\dot{\psi }}{v_{x}}$$

the following state-space model can be derived.

(12) $$\frac{d}{dt}\left[\begin{array}{c} y\\ \dot{y}\\ \psi \\ \dot{\psi } \end{array}\right]=\left[\begin{array}{cccc} 0 & 1 & 0 & 0\\ 0 & -2\frac{C_{\alpha f}\;+\;C_{\alpha r}}{mv_{x}} & 0 & -v_{x}-2\frac{C_{\alpha f}l_{f}\;-\;C_{\alpha r}l_{r}}{mv_{x}}\\ 0 & 0 & 0 & 1\\ 0 & -2\frac{C_{\alpha f}l_{f}\;+\;C_{\alpha r}l_{r}}{I_{z}v_{x}} & 0 & -2\frac{C_{\alpha f}l_{f}^{2}\;-\;C_{\alpha r}l_{r}^{2}}{I_{z}v_{x}} \end{array}\right]\left[\begin{array}{c} y\\ \dot{y}\\ \psi \\ \dot{\psi } \end{array}\right]+\left[\begin{array}{c} 0\\ \frac{2C_{\alpha f}}{m}\\ 0\\ \frac{2C_{\alpha f}l_{f}}{I_{z}} \end{array}\right]\delta$$

Since the objective is to design a steering control for automatic lane keeping in autonomous vehicles, it is useful to consider a state-space model where the state variables are the position error ($e_{1}$, i.e., the distance of the center of gravity of the vehicle from the centerline of the lane) and the orientation error with respect to the road ($e_{2}$). Then, by introducing the following error variables,

(13) $${\dot{e}}_{1}=\dot{y}+\int v_{x}{\dot{e}}_{2}dt,\;\;\;\;\;e_{2}=\psi -\psi_{des}$$

and by substituting them in Equation (11), the state-space model expressed in terms of the traking error variables can be obtained:

$$\frac{d}{dt}\left[\begin{array}{c} e_{1}\\ {\dot{e}}_{1}\\ e_{2}\\ {\dot{e}}_{2} \end{array}\right]=\left[\begin{array}{cccc} 0 & 1 & 0 & 0\\ 0 & -2\frac{C_{\alpha f}\;+\;C_{\alpha r}}{mv_{x}} & 2\frac{C_{\alpha f}\;+\;C_{\alpha r}}{m} & -2\frac{C_{\alpha f}l_{f}\;-\;C_{\alpha r}l_{r}}{mv_{x}}\\ 0 & 0 & 0 & 1\\ 0 & -2\frac{C_{\alpha f}l_{f}\;+\;C_{\alpha r}l_{r}}{I_{z}v_{x}} & 2\frac{C_{\alpha f}l_{f}\;+\;C_{\alpha r}l_{r}}{I_{z}} & -2\frac{C_{\alpha f}l_{f}^{2}\;-\;C_{\alpha r}l_{r}^{2}}{I_{z}v_{x}} \end{array}\right]\left[\begin{array}{c} e_{1}\\ {\dot{e}}_{1}\\ e_{2}\\ {\dot{e}}_{2} \end{array}\right]+\left[\begin{array}{c} 0\\ \frac{2C_{\alpha f}}{m}\\ 0\\ \frac{2C_{\alpha f}l_{f}}{I_{z}} \end{array}\right]\delta +$$

(14) $$+\left[\begin{array}{c} 0\\ \frac{2C_{\alpha f}}{m}\\ 0\\ \frac{2C_{\alpha f}l_{f}}{I_{z}} \end{array}\right]d+\left[\begin{array}{c} 0\\ -\frac{2C_{\alpha f}l_{f}-2C_{\alpha r}l_{r}}{mv_{x}}-v_{x}\\ 0\\ \frac{2C_{\alpha f}l_{f}^{2}-2C_{\alpha r}l_{r}^{2}}{I_{z}v_{x}} \end{array}\right]{\dot{\psi }}_{des}$$

where $\delta =\delta_{f}$ is the front steering angle and *d* is a disturbance signal. Furthermore, by assuming the following, it is possible to define the rate of change in the reference orientation of the vehicles as ${\dot{\psi }}_{des}=\frac{v_{x}}{R}$:

- The vehicle rides at longitudinal velocity $v_{x}$ on a curved road of radius *R*.
- *R* is sufficiently large so that the small angle assumption holds true.

Then, by integrating the latter expressions, it is possible to compute the reference yaw angle $\psi_{des}$.

**Remark** **1.** *The position of the vehicle in the global (world) coordinates (X,Y) can be computed from the body-fixed coordinates ($\dot{x},\dot{y}$) as follows:*

(15) $$\left\{\begin{array}{ccc} X & = & \int_{0}^{t}\left(v_{x}cos\left(\psi \right)-\dot{y}sin\left(\psi \right)\right)dt\\ Y & = & \int_{0}^{t}\left(v_{x}sin\left(\psi \right)+\dot{y}cos\left(\psi \right)\right)dt \end{array}\right.$$

*The lateral dynamics model given by Equation (14) is a function of the longitudinal vehicle speed $v_{x}$, which can be assumed to be a known varying parameter. The value of $v_{x}$ can be obtained through a standard feedback control law (e.g., PI control) in charge of providing the control action to track a desired speed $v_{x}^{d}$.*

## 3. Problem Statement and Controller Design

In general, a lane-following system is a control system that allows the vehicle to ride along a lane while maintaining a reference-set velocity and a safe distance from the preceding vehicle. This type of system combines longitudinal and lateral control for the following:

- Maintaining a driver-set velocity and to maintain a safe distance from the preceding car by adjusting the acceleration of the vehicle (*longitudinal controller*), and
- Ensuring the vehicle travels along the centerline of the lane by regulating the steering angle (*lateral controller*).

Within the above-described context, the control objective is to make the autonomous vehicle track a desired reference trajectory via regulation of the steering angle δ. To proceed to the controller design, the following compact state-space model of the vehicle lateral dynamics is introduced. We denote the following:

- $x=\;\left[e_{1},{\dot{e}}_{1},e_{2},{\dot{e}}_{2}\right]$, the plant state;
- $u=\;\left[\delta \right]$, the manipulable input;
- $\xi =\;\left[d,{\dot{\psi }}_{des}\right]$, the plant disturbance;
- y= x, the system output; and
- $z=\;\left[e_{1},e_{2},\right]$, the performance output signals.

Thus, the dynamic model (12) can be rewritten in the following parameter-dependent state-space representation:

(16) $$\Sigma :\left\{\begin{array}{c} \dot{x}\left(t\right)=A\left(v_{x}\left(t\right)\right)+Bu\left(t\right)+D_{d}\left(v_{x}\left(t\right)\right)\xi \left(t\right)\\ y(t)=Cx(t)\\ z\left(t\right)=C_{z}x\left(t\right) \end{array}\right.$$

where the system matrices depends on the varying parameter $v_{x}\left(t\right)$ that is assumed to be bounded as

(17) $${\underline{v}}_{x}\leq v_{x}\leq {\bar{v}}_{x}$$

The goal of the proposed controller is to reduce the position and the orientation error by controlling the steering angle. Another important issue in the controller design is related to minimization of the effects of the disturbance term ${\dot{\psi }}_{des}$ that causes the tracking errors to not converge to zero when the vehicle rides along a curve. Then, by assuming that the longitudinal velocity is bounded as described by Equation (17), it is possible to consider static state-feedback control laws as possible controller candidates,

(18) u(t)=Kx(t),

to be computed by solving a $H_{\infty }$ optimal control synthesis problem that can be formulated as a convex LMI optimization problem in the unknown matrices $X=X^{T}$ and *Y* [25]:

(19) $$\left\{\begin{array}{c} min_{X,Y,\gamma }\;\;\;\;\gamma \\ s.t.\\ \left[\begin{array}{ccc} A_{i}X+BY+XA_{i}^{T}+Y_{i}^{T}B_{i}^{T} & D_{d_{i}} & XC_{z}^{T}\\ D_{d_{i}}^{T} & -\gamma I & 0\\ C_{z}X & 0 & -\gamma I \end{array}\right]<0,\;\;i=1,2\\ X=X^{T}>0 \end{array}\right.$$

where each vertex i=1,2 corresponds to the system matrices computed for $v_{x}={\underline{v}}_{x}$ and $v_{x}={\bar{v}}_{x}$. If solvable, the optimal $H_{\infty }$ control gain is given by $K=YX^{-1}$.

## 4. Simulations

**Figure 12:**
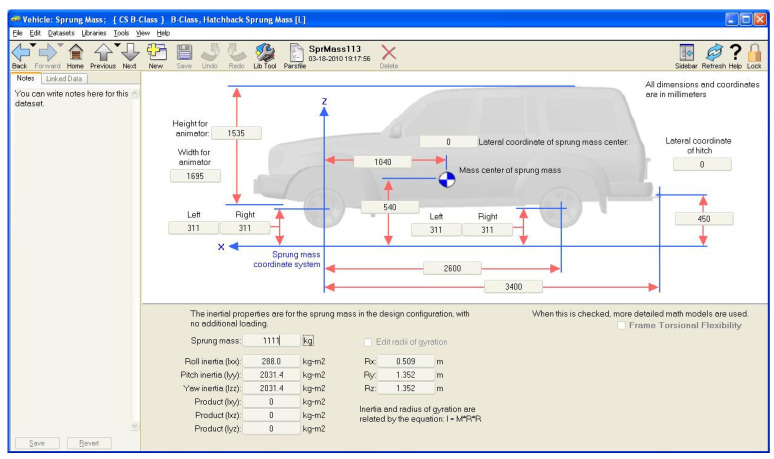
Carsim 8: vehicle parameter configuration.

**Figure 13:**
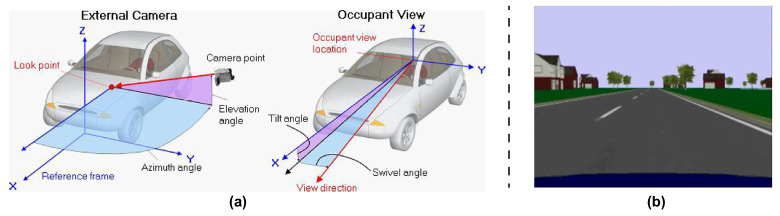
Carsim 8: camera point of view configuration (**a**) and camera front view (**b**).

**Figure 14:**
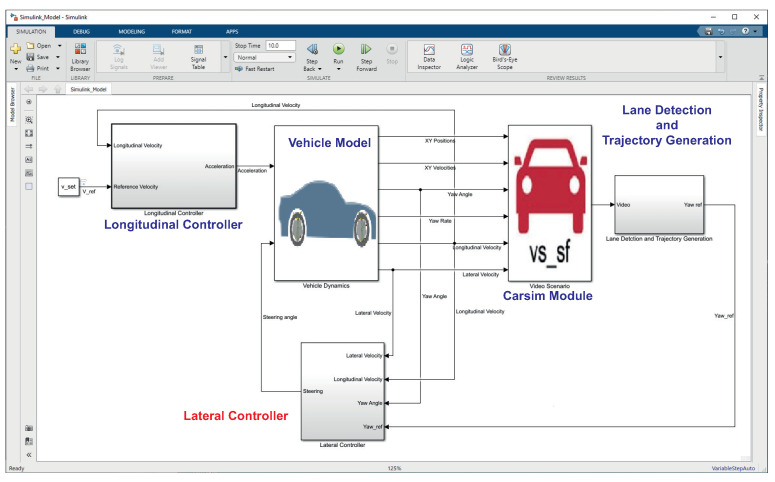
Co-simulation environment.

In order to verify the effectiveness of the proposed approach, some simulations have been undertaken under different guidance scenarios. In this respect, in order to consider realistic driving scenarios, a co-simulation environment based on the joint use of the MATLAB/Simulink *©* and Carsim 8 *©* packages was set up. The Carsim 8 software allows one to simulate and test autonomous driving systems by providing the possibility to add onboard vision systems as well as sensor fusion algorithms, path planning routines, and vehicle controllers. Visualization features also include the bird’s-eye-view plot, sensors (e.g., camera, radar, lidar, etc.) simulation and scope for sensor coverage, detections, and tracks (Figure 12 and Figure 13). The co-simulation environment model is depicted in Figure 14. Essentially, five modules have been included in this model:

1. The **Vehicle Model**, which models the longitudinal and lateral dynamics of the car. The inputs of the model are the longitudinal acceleration and the steering angle; the outputs of the model are the lateral and longitudinal velocities, the XY positions and velocities, the yaw angle, and the yaw rate of the vehicle;
2. The **Carsim Module**, which allows us to include all features related to the simulation of a driving scenario in the simulation environment. This module enables us to configure the vehicle parameters (Figure 12) and the camera point of view (Figure 13a). All of the outputs of the vehicle model are inputs of this module; the outputs of the module is a video containing the current scene (Figure 13b);
3. The **Longitudinal Controller**, which implements the control of longitudinal vehicle speed ($v_{x}$) through a PI controller. It computes the acceleration and deceleration commands on the basis of the current reference longitudinal speed. In particular, the controller implements the Stanley method, for which the details can be found in [14].
4. The **Lane Detection and Trajectory Generation Module**, which implements the algorithm reported in Section 2.2 and provides an estimation of the reference yaw angle;
5. The **Lateral Controller**, which implements the $H_{\infty }$ controller described in Section 3 and provides the steering angle command to the vehicle model. The control algorithm was designed in Matlab/Simulink, and the optimization problem (19) was solved using the SeDuMi Matlab toolbox.

The value reported in Table 2 were used as vehicle parameters.

**Figure 15:**
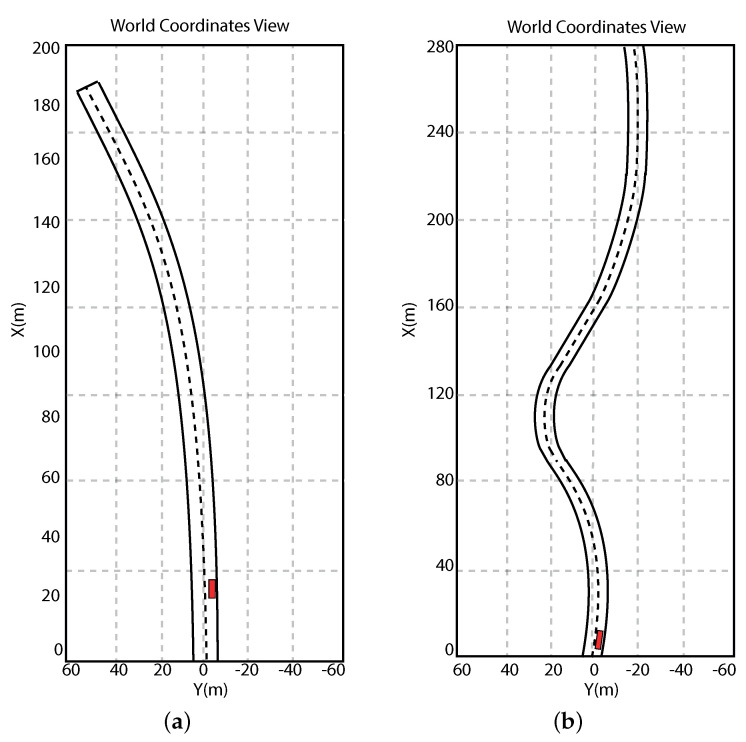
Simulation scenarios: scenario 1 (**a**) and scenario 2 (**b**).

Furthermore, in all scenarios accounted for (Figure 15), we considered longitudinal speed always in the range $5\leq v_{x}\leq 30$ m/s.

### 4.1. Scenario 1

The first simulation scenario refers to a driving condition where the autonomous vehicle must turn left on a road. Details about this scenario are reported in Figure 15a. In this condition, the autonomous vehicle must be able to perform the necessary actions allowing the car to ride on the road without taking risks. Then, the onboard electronic devices and the electronic control unit must accomplish the following tasks:

- Estimate the road lane;
- Compute the reference trajectory; and
- Perform the control actions thanks to which the vehicle can follow the computed trajectory.

**Figure 16:**
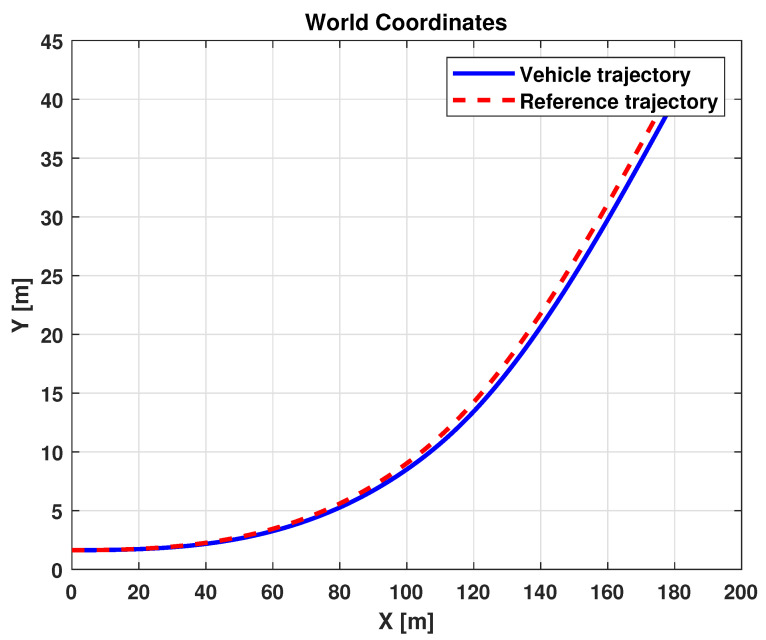
Simulation scenario 1: vehicle and reference trajectories.

**Figure 17:**
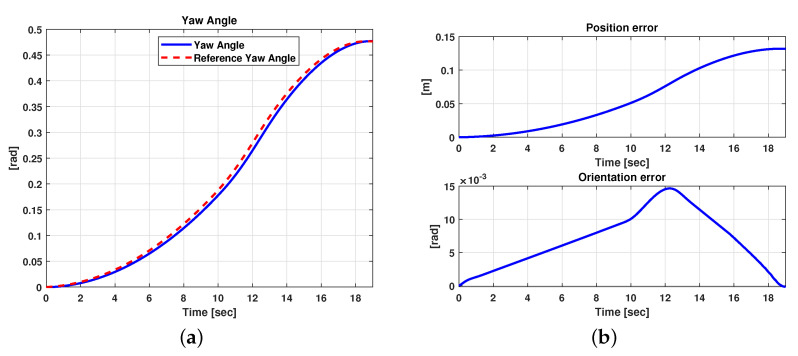
Simulation scenario 1: comparison between reference and measured yaw angles (**a**) and position and orientation errors (**b**).

The results related to this scenario are reported in Figure 16 and Figure 17. In particular, Figure 16 shows a comparison between the reference and the vehicle trajectories in the world coordinates. From this figure, it is evident that the proposed algorithm allows for good performance to be achieved in terms of trajectory followed. This result is also evident in Figure 17a,b, where a comparison between the reference and the measured yaw angle (a) and the position and orientation errors (b) is reported. A more quantitative comparison is reported in Table 3, where the error variables (Equation (13)) averaged along all the simulation time steps are reported.

### 4.2. Scenario 2

**Figure 18:**
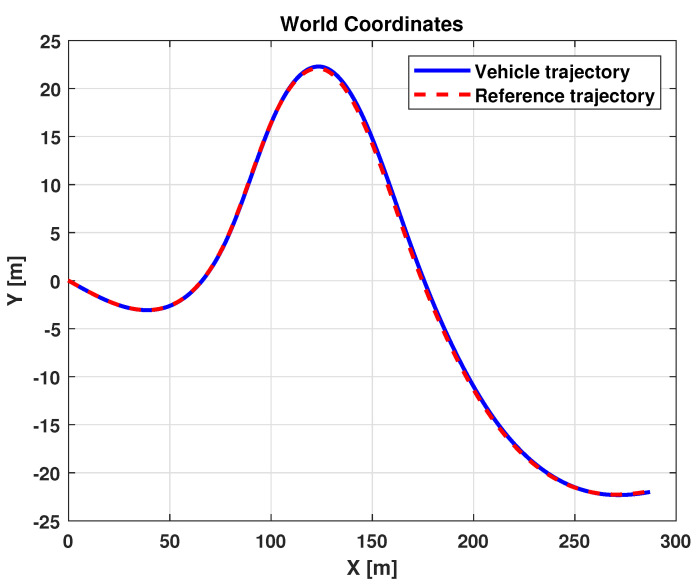
Simulation scenario 2: vehicle and reference trajectories.

**Figure 19:**
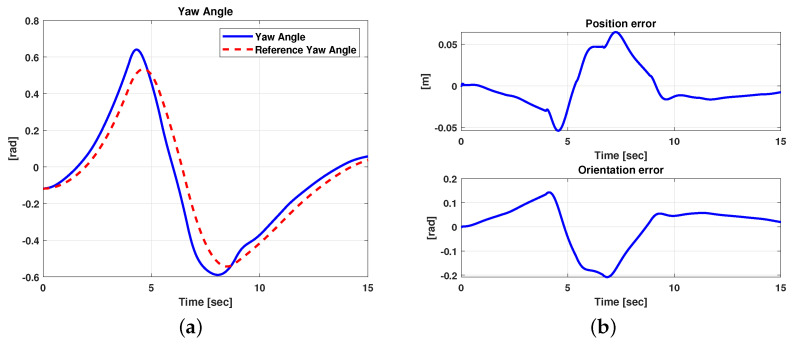
Simulation scenario 2: comparison between reference and measured yaw angles (**a**) and position and orientation errors (**b**).

In order to test the proposed method in a more complex driving condition, the scenario depicted in Figure 15b was taken into account. This scenario describes a driving condition where three consecutive road curves must be faced. The results related to this second scenario are reported in Figure 18 and Figure 19. It is evident that, in this case, the controller allows for good performance to be achieved in terms of the trajectory followed. Furthermore, as expected, Figure 19 shows small position and orientation errors, which tend toward zero when the curve ends and a straight road begins. As for the previous scenario, a quantitative performance evaluation was performed In this respect, Table 4 reports the error variables (Equation (13)) averaged along all the simulation time steps.

**Remark** **2.**  *Video clips related to validation of the lane-detection algorithm in a real scenario and to the assessment of the steering control algorithm can be found, respectively, at the following links:*

- *Lane detection algorithm in a real application scenario: https://youtu.be/4mcSdDFoivU (accessed on 5 June 2021).*
- *Steering control test in the co-simulation environment: https://youtu.be/CNrHAG6a4QU (accessed on 5 June 2021).*

## 5. Conclusions

In this paper, a procedure for lateral control of an autonomous vehicle was developed. The proposed approach consists of the design of a controller that is robust with respect to disturbance and variations in longitudinal vehicle speed. The control law was designed by solving a convex optimization problem and allows for a good trajectory-following performance to be achieved with low online computations. In fact, the optimization problem used for the synthesis is solved offline and only the computed controller gain enters the state-feedback control architecture. The simulations undertaken considered different driving conditions, and the results demonstrate the robustness of the designed control law. Despite these promising results, further efforts must be dedicated to improving the design of both longitudinal and lateral controllers in order to provide an integrated solution. In fact, this work focused on the design of a lateral control based on the assumption of an a priori knowledge of the longitudinal speed. Moreover, the approach only addresses how to eliminate orientation and position errors according to the desired path but does not take into account coupling with the longitudinal dynamics. In this respect, future work will address the design of a coupled lateral and longitudinal controller taking into account the linear parameter varying (LPV) framework as a possible solution [33]. The choice of the LPV framework is convenient in that it allows one use a single model to globally describe the dynamics of a nonlinear system subject to transitions between different working points and conditions, related to changes of some fundamental system parameters [34] and amenable for direct use for control synthesis purposes.

## Figures and Tables

**Table 1:** Camera parameters.

Parameter	Description	Value
**X(m), Y(m)**	*X*-axis and *Y*-axis positions of the camera in the vehicle coordinate system.	[0.83, 3.8]
**Height(m)**	Height of the camera above the ground.	1.2 m
**Focal length (X,Y)**	Horizontal and vertical point at which the camera is in focus.	[0.83, 6.2]
**Image Height and Width**	Horizontal and vertical point camera camera resolution (in pixel).	752 × 480
**Principal Point X and Y**	Horizontal and vertical image center (in pixel).	[376, 240]
**Update Interval**	Camera updating frequency.	61 FPS

**Table 2:** Vehicle parameters.

Parameter	Value	Description
*M*	1.575 Kg	Vehicle mass
$I_{z}$	2.875[1.2]	Inertia
$l_{f}$	1.2 m	Distance of the front tire from the vehicle center of gravity
$l_{r}$	1.2 m	Distance of the rear tire from the vehicle center of gravity
$C_{\alpha f}$	19.000 N/rad	Cornering stiffness of front tire
$C_{\alpha r}$	33.000 N/rad	Cornering stiffness of rear tire

**Table 3:** Scenario 1: relative errors.

	$e_{1}$ (%)	$e_{2}$ (%)
Scenario 1	4.46	5.79

**Table 4:** Scenario 2: relative errors.

	$e_{1}$[%]	$e_{2}$[%]
Scenario 2	5.06	6.19

## Data Availability

Not applicable.
